# On the role of the prefrontal cortex in fatigue effects on cognitive flexibility - a system neurophysiological approach

**DOI:** 10.1038/s41598-018-24834-w

**Published:** 2018-04-23

**Authors:** Vanessa A. Petruo, Moritz Mückschel, Christian Beste

**Affiliations:** Cognitive Neurophysiology, Department of Child and Adolescent Psychiatry, Faculty of Medicine of the TU Dresden, Dresden, Germany

## Abstract

Demanding tasks like cognitive flexibility show time-related deterioration of performance (i.e. fatigability effects). Fatigability has been associated with structural and functional properties of the prefrontal cortex. However, the electrophysiological underpinnings of these processes are not well understood. We examined n = 34 healthy participants with a task switching paradigm in which switches were either signaled by cues or needed to be maintained by working memory processes. We analyzed event-related potentials (ERPs) and performed residue iteration decomposition (RIDE) to account for effects of fatigue on intra-individual variability of neurophysiological data. This was combined with source localization methods. We show that task switching is affected by time on task (TOT) effects mostly when working memory processes are needed. On a neurophysiological level, this effect could not be observed in standard ERPs, but only after accounting for intra-individual variability using RIDE. The RIDE data suggests that during task switching, fatigability specifically affects response recoding processes that are associated with functions of the middle frontal gyrus (MFG; BA10). The results underline propositions of the ‘opportunity cost model’, which states that fatigability effects of executive functions depend on the degree to which tasks engage similar prefrontal regions - in this case working memory and task switching mechanisms.

## Introduction

In real-life situations, goal-directed behavior depends on control mechanisms associated with the prefrontal cortex^1,2^. However, prolonged continuous performance of cognitively demanding tasks, like for example doing complicated mental arithmetic compared to simply recognizing the individual digits constituting the arithmetic problem, induces cognitive fatigue and is associated with a time-related deterioration of performance, the degree of which is referred to cognitive fatigability^3^. Typically, cognitive fatigue is described as a decline of cognitive performances including information processing, working memory processes, planning, adequately preparing responses, sustained attention, ignoring irrelevant information and so on^3,4^. It must be distinguished from physical fatigue that involves the over-taxation of the muscular or cardiovascular system^5^. Additionally, the associated subjectively perceived tiredness or exhaustion cannot be compensated by an everyday recovery. Importantly, different chronic inflammatory diseases like multiple sclerosis (MS) and inflammatory bowel diseases (IBD) are severely affected by fatigability^6–9^. However, our study focuses on time-on-task (TOT) effects in healthy participants. TOT-related effects are assumed to be gradual and cumulative in nature^10–14^. Several lines of evidence reveal that cognitively demanding (effortful) tasks requiring cognitive control functions show mental fatigue effects^3,4,13^. In the last years, fatigability effects have attracted considerable interest and functional imaging studies have shown that declines in performance are related to changes in fronto-parietal networks and connected regions in the thalamus and the striatum^15–18^. Studies using network analyses of fMRI data^5,18^ or EEG data^19^ suggest that long-range connectivity is altered when fatigue effects emerge. A right > left hemispheric asymmetry in frontal connectivity at the beginning of a task for example, was reversed to a left > right asymmetry over the course of the task^5^. However, to explain effects of time-related deterioration of performance (i.e. cognitive fatigability), the ‘opportunity cost model’^3^ has been proposed focusing on structural and functional features of the prefrontal cortex. This model proposes that when subjects perform a task, the costs of doing so are represented. Over time, the estimate of the expected benefit to allocate full processing resources to the task declines so that the representations of costs grow with the time on task^3^. These fatigability effects have been suggested to particularly occur in tasks requiring executive control functions and associated structures in the prefrontal cortex^3,13^. The reason is that the prefrontal cortex is subject to simultaneity constraints; i.e. there is a capacity limitation to the number of computational operations the prefrontal cortex can engage in at any given time. According to Kurzban *et al*.^3^, there will be large opportunity costs to performing tasks that recruit the prefrontal cortex, because all of the tasks cannot be performed simultaneously as they require the same prefrontal processes^3^.

However, quite a few cognitive sub-processes may underlie the effects of fatigue on prefrontal cortical functions and behavioral control. They range from perceptual and attentional selection processes to cognitive control and decision-making processes and to motor processes^3,4^. It is therefore important to dissociate between these processes to examine what functions and sub-processes associated with the prefrontal cortex are particularly prone to fatigability effects. The more specific fatigue-based restrictions are provided due to the precise determination of impaired cognitive sub-processes, the higher the likeliness to pave a way to potential compensatory measures. The advantage of electrophysiological (EEG) techniques, event-related potentials (ERPs) and related methods is that these methods allow for a separation of different cognitive neurophysiological sub-processes that contribute to information processing on the basis of their temporal occurrence. In combination with source localization techniques, this makes it possible to examine the functional neuroanatomical network that is modulated. This is the goal of the current study in which we focus on fatigability effects on cognitive flexibility – a major instance of executive control associated with the prefrontal cortex^1^.

Cognitive flexibility mechanisms can be examined during task switching. Switching between responses leads to an increase in processing times as compared to non-switch task response times. This phenomenon is known as switch costs^20^. Since task switching processes are well-known to depend on prefrontal cortical networks^1^, it is very likely that time on task (fatigability) compromises this important faculty of cognitive control. As mentioned, the strength of fatigue effects however depends on the degree to which tasks engage similar prefrontal regions^3^. Within the context of task switching, it has been shown that switching becomes complicated when working memory processes, as opposed to external cues, have to be used to initiate switching processes. This is typically the case when subjects have to remember the rules and the time points of rule changes necessary to switch responses^21–25^. Therefore, we hypothesized that fatigability effects are most prominent when cognitive flexibility is implemented via working memory processes, because these also impinge on prefrontal networks^1^. However, there is a multitude of cognitive sub-processes (e.g. response reconfiguration processes) and associated neurophysiological mechanisms (e.g. activation differences in a response selection time frame) that may underlie such effects (e.g. decreased flexibility). In the current study, we hence examined this hypothesis with a system neurophysiological approach that combines high-density EEG recordings with source localization approaches.

When using EEG methods, switching mechanisms are reflected by the N2 and P3 event-related potential (ERP) components: During switching, mechanisms related to the resolution of conflict between simultaneously active stimulus-response mappings are reflected by the N2 event-related potential (ERP)^21,26,27^. Similarly, processes related to the implementation of a switching task-set lead to decreases in the P3 during efficient switching^11,21,26–33^. It is therefore possible that one or both of these processes are modulated by fatigability effects in memory-based task switching.

However, an important aspect that must be considered when investigating fatigability effects on the neurophysiological level relates to the strong intra-individual variability of neurophysiological processes during memory-based switching^22–25,34^. It has been shown that during memory-based switching, reliable neurophysiological correlates of cognitive sub-processes can best be detected when accounting for the issue of intra-individual variability^22^. This is of special importance in the context of fatigability, as intra-individual variability and its change over time is a key metric for defining performance-based cognitive fatigability^35^. Yet, it has been shown that the ERP method can only yield accurate insights into the neurophysiological processes of cognitive functions when there is little intra-individual variability^36,37^. We therefore hypothesized to only obtain reliable insights in the neurophysiology and associated functional neuroanatomy of fatigue effects during task switching when intra-individual variability is taken into account. To do so, we applied residue iteration decomposition (RIDE) on the EEG data^36,37^. Besides accounting for issues of intra-individual variability in neurophysiological data, this method also allows to dissociate different informational contents intermingled in the neurophysiological signal^38–40^. This is important because ERP components reflecting task switching sub-processes (i.e. N2 and P3) are known to reflect a mixture of stimulus-related and response-related processes^41–43^ that become dissociable after applying RIDE^44^: Stimulus and response related processes are reflected by distinct “clusters”; i.e. the S-cluster (reflecting perceptional and attentional processes) and the R-cluster (reflecting motor response related processes). An additional third cluster, the C-cluster, has been suggested to refer to intermediate processes between S and R (like stimulus evaluation and response selection)^36^. Using RIDE, it has been shown that the C-cluster and areas in the inferior parietal cortex and the temporo-parietal junction (TPJ; BA40) reflect processes in memory-based task switching^40^. Because effects of fatigability mainly affect prefrontal cortical functions^3^, it is unlikely that processes reflected by the C-cluster are affected by fatigue effects. Instead, we hypothesized that the R-cluster may reflect fatigue effects as several lines of evidence show that response-based interference processes play an important role in task switching^45^. Since these processes have been associated with the function of the lateral prefrontal cortex^46^ and fatigability particularly affects prefrontal circuits^3^ response-based interference processes may be modulated in particular. Taken together, it is possible that reliable fatigue effects can only be obtained at the neurophysiological level after applying RIDE, i.e. when intra-individual variability is accounted for. Therefore, it is possible that no reliable effects are obtained at the standard ERP level, but after applying RIDE. After applying RIDE it is likely that the R-cluster data is modulated and reflects interactive effects as expected for the behavioral data.

## Results

### Behavioral data

#### Accuracy

The repeated-measures ANOVA revealed a significant main effect for the factor “repetition-switch” (F_1,33_ = 32.96; p < 0.001; η_p_^2^ = 0.500) with higher accuracy for repetition (95.38 ± 0.43) than switch trials (93.82 ± 0.59). Moreover, a significant interaction of “repetition-switch × blocks” was detected (F_2,66_ = 3.67; p = 0.035; η_p_^2^ = 0.100). For the post-hoc tests, the switch costs (switches trials minus repetition trials) were calculated for every block. There were higher switch costs in block 3 (2.12 ± 1.83) than in block 1 (1.08 ± 2.29) (t_34_ = 2.35; p = 0.025). All other comparisons were not significant (all p > 0.10). All other main or interaction effects were not significant (all F < 3.50; p > 0.07).

#### Response times

**Figure 1:**
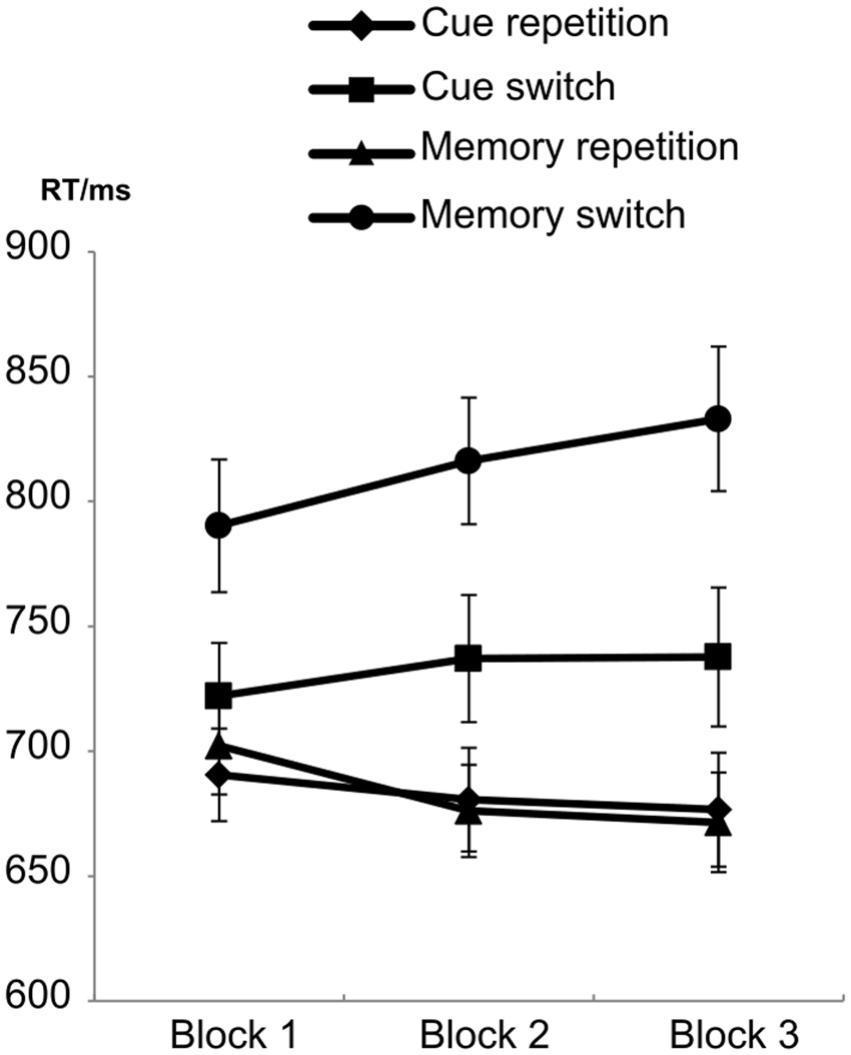
Reaction times (RTs) in milliseconds in the three experimental blocks (block 1 till block 3). The means and standard errors of the different experimental conditions “cue repetition” (diamond), “cue switch” (square), “memory repetition” (triangle), and “memory switch” (circle) are shown.

The repeated-measures ANOVA showed a significant main effect of “repetition-switch” (F_1,33_ = 110.46; p < 0.001; η_p_^2^ = 0.770) with faster reaction times (RTs) for repetition (682 ± 18) than for switch trials (772 ± 24). Additionally, a main effect of “trials” was evident (F_1,33_ = 19.39; p < 0.001; η_p_^2^ = 0.370), showing faster RTs in the cue-based trials (707 ± 21) than in the memory-based trials (748 ± 21). Furthermore, significant interactions of “trials × repetition-switch” (F_1,33_ = 77.82; p < 0.001; η_p_^2^ = 0.702), “repetition-switch × blocks” (F_2,66_ = 30.94; p < 0.001; η_p_^2^ = 0.484) and “trials × repetition-switch × blocks” (F_2,66_ = 5.09; p = 0.009; η_p_^2^ = 0.134) were evident. The most important and complex interaction “trials × repetition-switch × blocks” was further analyzed and is illustrated in Fig. 1.

Post-hoc tests demonstrated that an interaction of “repetition-switch × blocks” was evident in both cue-based (F_2,66_ = 6.93; p = 0.002; η_p_^2^ = 0.174) and memory-based trials (F_2,66_ = 24.64; p < 0.001; η_p_^2^ = 0.428). However, the effect size was larger in memory-based trials showing that the “trials × repetition-switch × blocks” interaction is driven by the trials factor. In the repetition condition of memory-based trials, as RTs got faster from block 1 to block 3 (F_2,66_ = 5.20; p = 0.011; η_p_^2^ = 0.136): Block 1 differed significantly from block 2 (p < 0.01) and from block 3 (p < 0.02). In the switching condition of memory-based trials, RTs increased from block 1 to block 3 (F_2,66_ = 4.92; p = 0.011; η_p_^2^ = 0.130). Again, block 1 differed significantly from block 2 (p < 0.05) and from block 3 (p < 0.01). The effect sizes for switches and repetitions were virtually the same (i.e. η_p_^2^ = 0.136 in repetition and η_p_^2^ = 0.130 in switches). Therefore, the increase in switch costs (switches minus repetitions) is equally driven by changes in switches and repetitions. By calculating the switching costs (switches minus repetitions), it was shown that these increased from the first (59 ± 43) to the second (100 ± 51) to the third block (111 ± 67) (F_2,60_ = 29.49; p < 0.001; η_p_^2^ = 0.496) with the first block differing from block 2 (p < 0.001) and from block 3 (p < 0.001).

#### RT intra-individual variability

We also analyzed the intra-individual variability in RTs. This was done by running a repeated measures ANOVA on the RT standard deviations of each subject. The ANOVA revealed a main effect of “repetition-switch” (F_1,33_ = 11.15; p = 0.002; η_p_^2^ = 0.253), showing higher SDs for switch (307 ± 12) than for repetition trials (286 ± 13). The main effect of “trials” showed that SDs were larger in memory-based (328 ± 12) than in cue-based trials (265 ± 11) (F_1,33_ = 126.25; p < 0.001; η_p_^2^ = 0.793). The main effect of “block” (F_1,33_ = 5.65; p = 0.007; η_p_^2^ = 0.146) showed that SDs were larger in the third (304 ± 14) and the second block (300 ± 12), as compared to the first block (285 ± 13) (all p < 0.01).

#### Neurophysiological data

As shown in the behavioral data (RTs), switch costs increased from the first to the second block. This effect was stronger in memory-based trials than in cue-based trials and changes in repetitions and switches contributed equally to this effect; i.e. the effect sizes for switches and repetitions were virtually the same; i.e. η_p_^2^ = 0.136 in repetition and η_p_^2^ = 0.130 in switches (see above). Therefore, only difference waves between switches and repetitions were considered for the neurophysiological data analysis. The ANOVA models used are outlined in the statistics section.

#### Standard ERPs

**Figure 2:**
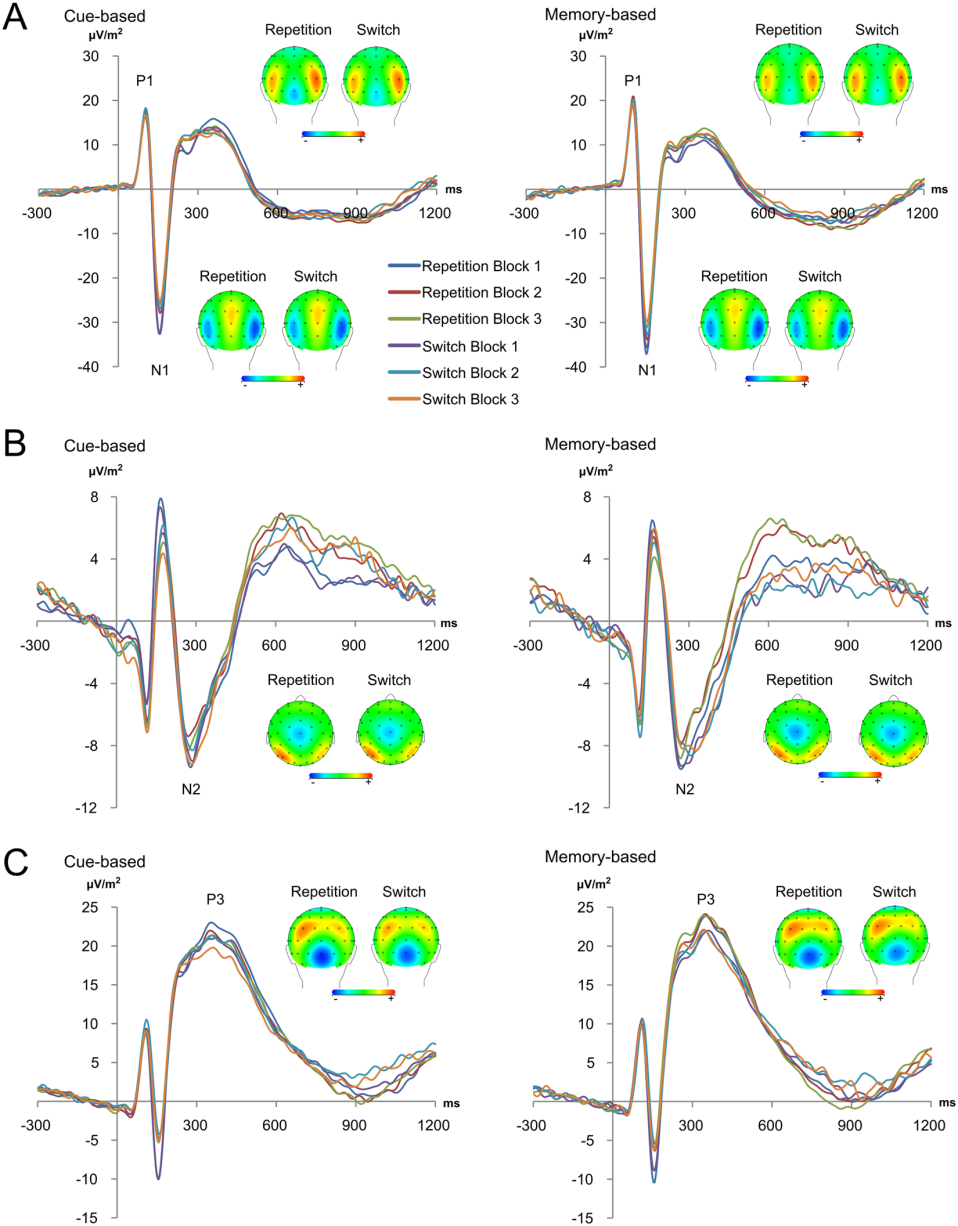
Event-related potential (ERP) components in the cue-based (left) and memory-based (right) condition. (**A**) The P1 (~100 to 110 ms) and N1 (~150 to 160 ms) ERP-components are shown for electrode P7 and P8. The N2 ERP-component at electrode Cz is shown in (**B**). The P3 ERP-component (~320 to 460 ms) is shown at electrodes P3 and P4 (**C**). Time point zero denotes the time point of target stimulus presentation. Additionally, scalp topographies of repetition versus switch trials of the two different experimental conditions are shown at the maximum peak for respective ERPs. In the topographies, positivity is shown in red and negativity in blue.

The P1, N1, N2 and P3 ERP difference waves (mean amplitude differences between repetition and switch trials) are shown in Fig. 2. No significant main or interaction effects were detected for P1 (all F < 2.13; p > 0.16) and N1 amplitudes (all F < 3.58; p > 0.06). Moreover, no significant main or interaction effects were shown for N2 amplitudes (all F < 1.45; p > 0.20). An interaction of “trials × blocks” was evident for P3 amplitudes (F_2,66_ = 3.22; p = 0.05; η_p_^2^ = 0.089), but post-hoc tests did not survive Bonferroni correction (p > 0.09). An interaction of “blocks × electrodes (P3 vs. P4)” was also detected for P3 amplitudes (F_2,66_ = 4.17; p = 0.024; η_p_^2^ = 0.112). Yet, post-hoc tests did again not survive Bonferroni correction (all p > 0.09). The results were unchanged when other time intervals for data quantification were used (i.e. when the same time windows as in the RIDE data analyses were used).

#### Residual iteration decomposition (RIDE)

**Figure 3:**
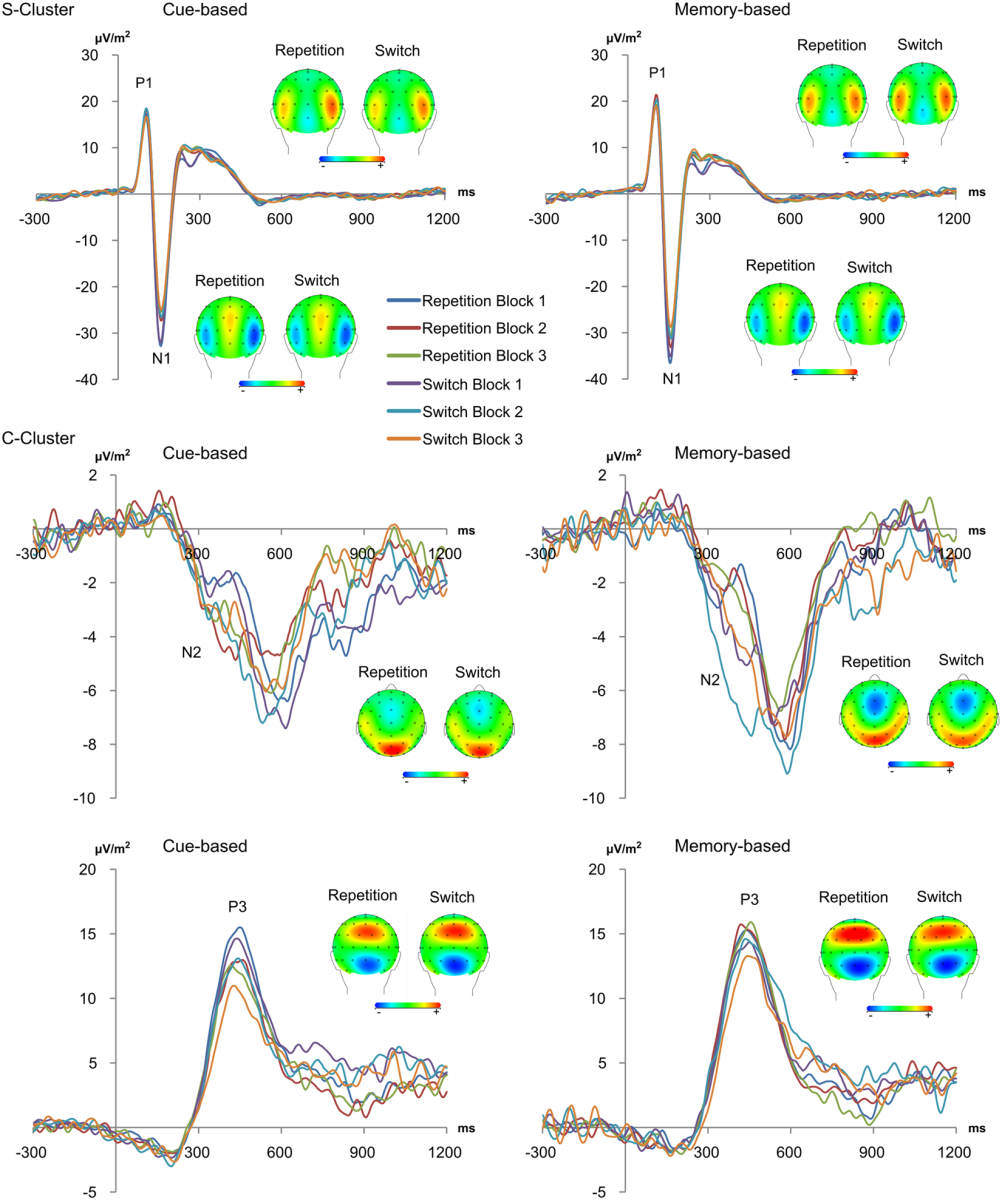
The S-cluster and C-cluster from the RIDE analyses are presented for the cue-based (left) and memory-based (right) condition. The S-cluster (top) reflects activity in the P1 (~100 to 110 ms) and N1 (~150 to 160 ms) time window at electrode P7 and P8. The C-cluster reflects activity in the N2 time window at electrode FCz (middle part of the figure) in the cue-based trials (left) and the memory-based trials (right). Moreover, the activity in the P3 time window was evident (lower part of the figure). Scalp topographies of the four different experimental conditions are included, showing the respective RIDE topography at the maximum of respective peaks in the different time windows. In the topographies, positivity is shown in red and negativity in blue.

As expected (refer introduction), no reliable effects could be detected using a standard ERP analyses. Since this lack of effects may reflect issues related to the intra-individual variability, residue iteration decomposition (RIDE) was applied. The S-cluster differences waves are shown in Fig. 3. A P1 and an N1 component can be seen. The repeated measures ANOVAs for the P1 and N1 peaks in the S-cluster revealed no significant main or interaction effects (all F < 3.32; p > 0.07).

The C-cluster difference waves are also shown in Fig. 3. The C-cluster included a peak in the N2 time window and a peak in the P3 time window. For the peak in the N2 time window, a repeated-measures ANOVA revealed a main effect of “trials” (F_1,33_ = 4.86; p = 0.035; η_p_^2^ = 0.128), with higher difference wave amplitudes in memory-based trials (−2.88 µV/m^2^ ± 0.60) compared to cue-based trials (−0.86 µV/m^2^ ± 0.69). A main effect of “blocks” (F_2,66_ = 4.52; p = 0.019; η_p_^2^ = 0.120) revealed the highest difference wave amplitudes in the second block (−3.10 µV/m^2^ ± 0.57), as compared to the first block (−2.36 µV/m^2^ ± 0.71) and the third block (−0.15 µV/m^2^ ± 0.91). However, no interaction of “trials × blocks” was detected (F < 0.78; p > 0.5). For the peak in the P3 time window, the repeated-measures ANOVA revealed no significant main or interaction effects (all F < 1.74; p > 0.18).

**Figure 4:**
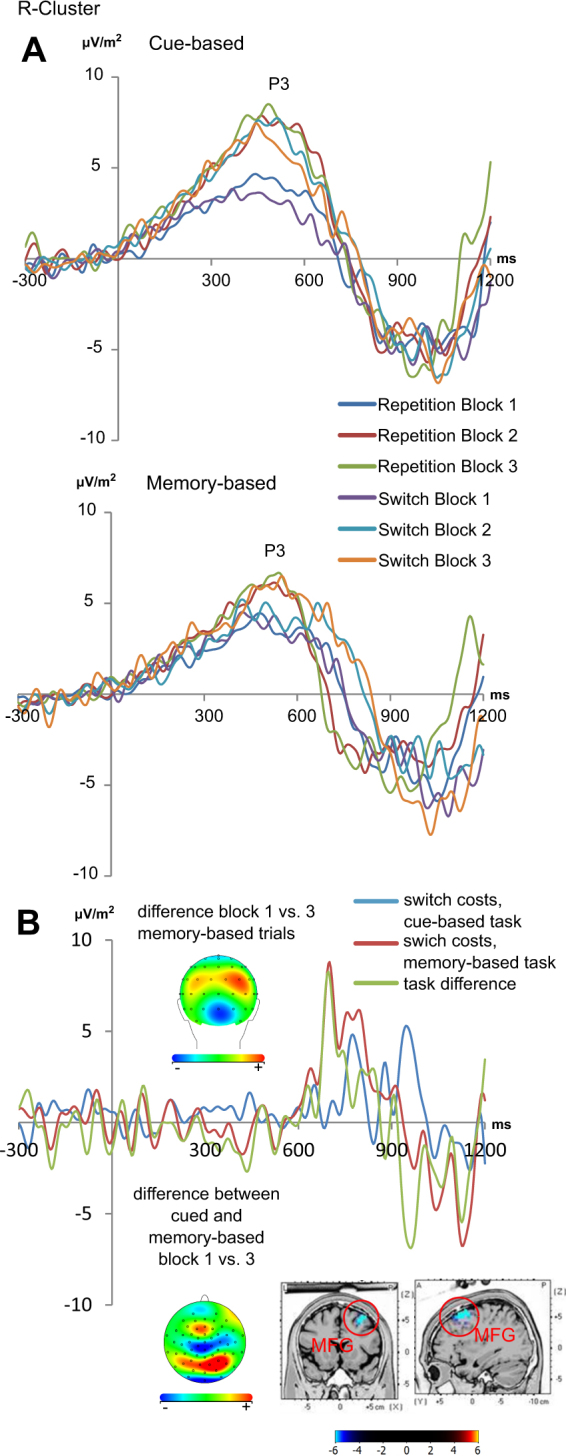
The R-cluster from the RIDE analyses is presented. (**A**) The R-cluster shows activity in the P3 time window at electrodes P4 and PO2 for the cue-based (upper part) and memory-based trials (lower part). As our behavioral results showed that switch costs (switching minus repeating trials) were modulated over time (experimental blocks), the switch costs (i.e. differences between the first and third block) are shown separately for cue-based trials (blue) and memory-based trials (red). Significant modulations of switch costs were shown only in the memory-based trials. Topographies for the differences between the first and third block at the maximum peak are shown for the memory-based trials in the upper part of the figure and for cued trials in the lower part of the figure. In the topographies, positivity is shown in red and negativity in blue. The difference in switch costs between the tasks (memory-based minus cued trials) is presented in green including the all-in-one scalp topography plot. Time point zero denotes the time point of target stimulus presentation. The sLORETA results show the source of the neurophysiological signal in this time range in BA10 (corrected for multiple comparisons). The sLORETA colour scale shows critical t-values.

The R-cluster difference wave data is shown in Fig. 4. As shown in the scalp topography plots, it was maximal over parietal electrodes (i.e. P4 and PO2). A repeated-measures ANOVA with the factors “trials”, “blocks” and “electrodes” (P4 and PO2)” revealed a main effect of “trials” (F_1,33_ = 5.92; p = 0.021; η_p_^2^ = 0.152) with larger difference wave amplitudes in memory-based trials (2.95 µV/m^2^ ± 1.22) than in cue-based trials (−0.87 µV/m^2^ ± 0.72). Furthermore, a main effect of “blocks” was shown (F_2,66_ = 7.20; p = 0.002; η_p_^2^ = 0.179), revealing the largest difference wave amplitudes in the third block (2.75 µV/m^2^ ± 0.98), followed by the second block (1.93 µV/m^2^ ± 1.07), and the first block (−1.58 µV/m^2^ ± 0.74). Most importantly, the interaction of “trials × blocks” was significant (F_2,66_ = 5.34; p = 0.011; η_p_^2^ = 0.139). Post-hoc tests found differences between the difference wave amplitudes only in memory-based trials (F_2,66_ = 11.67; p < 0.001; η_p_^2^ = 0.26), with significant differences between the first and the second block (p < 0.01) and between the first and the third block (p < 0.001): R-cluster amplitudes were larger in the second (3.76 µV/m^2^ ± 1.76) and third block (6.73 µV/m^2^ ± 1.81), compared to the first block (−1.65 µV/m^2^ ± 1.11). The sLORETA analysis showed that this effect was associated with activation differences in the right middle frontal gyrus (MFG, BA10) (first block < second block; first block < third block). In contrast to this, R-cluster difference wave amplitudes in the cue-based trials were not different between blocks (F < 0.50; p > 0.50). Taken together, a fatigue effect that is in line with the behavioral data was only evident in the R-cluster data in the P3 time window, and was due to activation differences in the right middle frontal gyrus (BA10).

## Discussion

In the current study, we examined the neurophysiological and functional neuroanatomical basis of fatigability effects in task switching using EEG methods. We employed a task switching paradigm in which the need to switch was either signaled by cues, or needed to be triggered by working memory processes. This experimental variation was introduced because several lines of evidence have revealed that (i) especially cognitively demanding (effortful) tasks requiring executive control functions show mental fatigue effects ^3,4,13^ and (ii) that the degree to which performance is affected depends on the degree to which the tasks engage similar prefrontal regions^3^. It has been shown that working memory processes can increase demands on task switching processes^22,23,25,34,40^ and we hypothesized switching fatigability effects are therefore particularly evident in memory-based tasks.

In fact, the behavioral results provide evidence that working memory-based cognitive flexibility processes are particularly affected by fatigue effects, as there was a selective increase in switch costs from block 1 to block 3 in the memory-based condition. In the cue-based condition, no block-related modulations were observed. The increase in switch costs (i.e. difference between switch and repetition trials) was constituted by modulations of RTs in both switch and repetition trials. The effect sizes show that increases in RTs in switching and decreases in RTs during repetition trials are equal.

The neurophysiological data provides insights into the nature of the cognitive sub-processes that are altered by fatigability. Using a standard analysis of ERPs, none of the examined ERP-components (i.e. P1, N1, N2 and P3) revealed an interaction in line with the behavioral data. For the P1 and N1 ERP components, this is an expected result since attentional selection processes are have not been shown to underlie modulations in motor task switching. However, effects on the N2 and/or the P3 ERP-component could have been expected because the N2/P3 ERP components have consistently been shown to be modulated in task switching and reflect conflict between simultaneously active stimulus-response mappings during response selection, or processes related to the implementation of a switching task-set, respectively^21,26,27,11,21,26–33^. Importantly and as outlined in the theoretical background of the introduction section, it has already been shown that reliable modulations of neurophysiological sub-processes during memory-based task switching can be detected best when accounting for intra-individual variability^40^. This is of particular relevance because intra-individual variability and its possible change over time are considered to reflect a key metric of fatigability effects^47^. Recent results have also shown that intra-individual variability in neurophysiological processes can be evident in the absence of strong intra-individual variability at the behavioral level^48^. Still, the reaction time data of this study shows that intra-individual variability did indeed increase through the course of the experiment (see analysis in section 3.1.3), which is in line with other findings^47^.

Against this background, it seems reasonable that effects reflecting the behavioral data were only obtained after applying RIDE on the EEG data. RIDE accounts for intra-individual variability^36^ and makes it possible to distinguish between different processes that are otherwise intermingled in standard ERPs^44^. It is important to mention that RIDE does not strip the signals from important content. Rather, one of the benefits of RIDE is that ERPs are decomposed into components clusters with different latency variabilities^37^ so that variance is not stripped away. Instead, the variance is used to ‘create’ reasonable component clusters, which ultimately reduces noise in the data. The consequence of this transformation is that RIDE-decomposed data are more sensitive for modulations at the neurophysiological level^48^. This is why we observed effects in the RIDE-decomposed data, but not the original ERP data.

We demonstrated that the S-cluster and the C-cluster do not reflect the interaction observed at the behavioral level. This suggests that processes of perceptual gating and attentional selection, which are reflected by the S-cluster^36^, are unlikely to underlie fatigability effects in memory-based task switching. The C-cluster has been suggested to resemble processes reflected by the P3 ERP-component^43^ and may reflect processes related to the updating of internal representations and task sets for response selection during memory-based task switching^24^. These processes were however not modulated by fatigability effects as the C-cluster showed no interactive effects in line with the behavioral data. The reason for this may be that the C-cluster has already been shown to be associated with the inferior parietal cortex (BA40) during memory-based task switching^24^. Yet, according to the opportunity cost model^3^, especially prefrontal cortical structures are susceptible to fatigue effects. It is therefore possible that processes reflected by the C-cluster are not modulated because these functions do not rely on mechanisms employing the prefrontal cortex, where effects of fatigability are most prominent^3,4^.

Interestingly, the R-cluster reflected fatigability effects in line with the behavioral data, thus suggesting that aspects of response-related processes during task switching are most likely and strongly modulated by fatigue effects. Indeed, several lines of evidence have shown that response-based interference processes play an important role in task switching^45^, especially when there are ‘bivalent responses’ and overlaps in ‘response sets’. Bivalent responses occur when identical motor responses are either used in different tasks, or for the same stimulus categories in a task switching experiment^45^. This was the case in the current study, as the response categories “numeric”, “parity” and “font size” overlapped; i.e. left and right hand responses were required in each of these categories (refer methods section). It has been suggested that such overlaps in response categories lead to interferences in task switches and therefore increase switch costs^49,50^. This response-related aspect of switching (and switch costs) is referred to as ‘response recoding process’^45,51^. It is possible that such response recoding processes are reflected in the R-cluster. The behavioral switch costs, as well as the switching effect in the R-cluster became larger across blocks in the memory-based condition than in the cue-based condition. It is possible that with increasing time spent on the memory-based task, the inference between response categories becomes increasingly difficult to dissolve at the level of motor responses, or response recoding. It has been suggested that response recoding processes are likely to be triggered by the response itself and therefore occur quite late during information processing^45^. In line with this, the effects observed in the R-cluster data occur ~700 ms after target stimulus presentation and thus in the time range of the RTs. This underlines the interpretation that the modulations seen in the R-cluster may reflect response recoding processes. Yet, modulations of response recoding processes only happen when working memory processes are used to guide switching. The sLORETA analysis suggests that such modulations of the R-cluster were due to modulations of neuronal activity in the right middle frontal gyrus (MFG; BA10) (contrast: block 1 < block 3). Previous results using fMRI suggest that response recoding processes are associated with the right lateral prefrontal cortex during task switching^46^. Such lateral prefrontal regions have also been shown to be important in response inhibition processes^52–58^. This is relevant in the context of this study/experiment because inhibitory control processes occur during task switching^59,60^. Especially bivalent responses and response recoding processes have been suggested to trigger mechanisms that inhibit competing response mappings^45^. Moreover, the MFG has been shown to become activated when demands on working memory processes are increased^61–65^. It is therefore possible that the reason why fatigue effects are mainly observed in the memory-based condition is because working memory and response recoding processes put high demands on neural processes in prefrontal networks, presumably the right middle frontal gyrus, and that this ultimately makes these prefrontal neural processes susceptible to time-on-task (fatigability) effects. Importantly, this interpretation is completely in line with assumptions of the opportunity cost model on fatigue effects by Kurzban *et al*.^3^. According to this account, opportunity costs leading to declines in task performance with increasing “time-on-task” are particularly high when the involved cognitive processes rely on prefrontal networks. Since there are capacity limitations to the number of computational operations that the prefrontal cortex can engage in at any given time, there are large opportunity costs to performing tasks recruiting the prefrontal cortex. Yet, this also implies that the degree to which performance is affected depends on the degree to which the tasks engage similar prefrontal regions^3^. Given this theoretical background, it seems reasonable that response recoding processes associated with prefrontal structures are only affected when working memory demands are increased, as switching and working memory processes presumably recruit overlapping areas in the lateral prefrontal cortex.

In summary, the study investigated the boundary conditions of fatigability effects on cognitive flexibility. The behavioral results show that task switching is affected by TOT effects, mainly when working memory processes are needed to control task switching. On a neurophysiological level, this effect could only be observed after accounting for intra-individual variability in the data. The neurophysiological data suggests that during task switching, fatigability specifically affects response recoding processes that are associated with functions of the middle frontal gyrus (MFG; BA10). The results underline the propositions of the opportunity cost model^3^, which states that fatigability affects functions of the prefrontal cortex and that the degree to which performance is affected depends on the degree to which the tasks engage similar prefrontal regions - in this case working memory and task switching mechanisms. Our findings provide new insights into the neurophysiological underpinnings on TOT effects underlying cognitive flexibility. TOT effects are strongly associated to fatigability effects occurring in a wide range of chronic diseases like MS and IBD.

## Materials and Methods

### Participants

The sample consisted of n = 34 healthy subjects (n = 21 females) between 20 and 29 years (M = 24.88; SD = 2.75). All participants were students and had at least completed 13 years of schooling. Moreover, all student participants were right-handed, had normal vision, and were non-smokers. None of them had any neurological or psychiatric disorders. All participants were treated according to the declaration of Helsinki^66^. The study and all experimental procedures were approved by the ethics committee of the TU Dresden. The methods were carried out in accordance with these regulations. Written informed consent was obtained from all participants.

### Procedure

All participants were invited to the EEG laboratory at 8 a.m. They were instructed not to drink coffee or other caffeinated beverages before the experiment. Instead of informing them about the investigation on mental fatigue, they were told that they served as control participants for a previous study. They were not informed that the switching task was going to be the only task they had to perform that day until briefly before the start of the paradigm. At this time point, they were also told that the task was divided into three blocks with duration of approximately 60 minutes per block. Subsequently, all participants answered questionnaires about their demographical data and self-reported psychological well-being. This included the german versions of the “Beck Depression Inventory (BDI)”^67^ and the “Fatigue Scale for Motor and Cognitive Functions (FSMC)”^68^. Both questionnaires, BDI (mean = 3.19, SD = 3.64) and FSMC (mean for cognitive scale = 18.29, SD = 6.32; mean for motor scale = 17.41, SD = 5.28; overall mean = 35.71, SD = 11.12) were below thresholds of pathological values. According to the guidelines of the BDI, total values of 0 to 63 are possible. Values below 11 are considered as inconspicuous. Values above 11 up to 17 are considered as mild to moderate forms of depression. Of pathological relevance are values above 18. While answering the questionnaire, the EEG caps were put on by the experimenter and the medical-technical assistant. Both, the completion of the questionnaires and the installing of the EEG caps took place in the test chamber. After offering a detailed explanation of the task rules, participants had to practice the task for at least 18 trials or until they and the experimenter felt that the task was fully understood. They were furthermore instructed to answer as quickly and accurately as possible throughout the entirety of the upcoming experiment. Once the experiment had started, participants were given breaks of 7 minutes between the first and the second block and between the second and the third block. In these breaks, participants were allowed to drink some water. The entire duration of the experiment was 4 hours and included the preparation time and the time needed after the pure testing time (e. g. hair washing). After the experiment, participants were debriefed and received an expense allowance of 45 Euro.

### Stimuli and task

**Figure 5:**
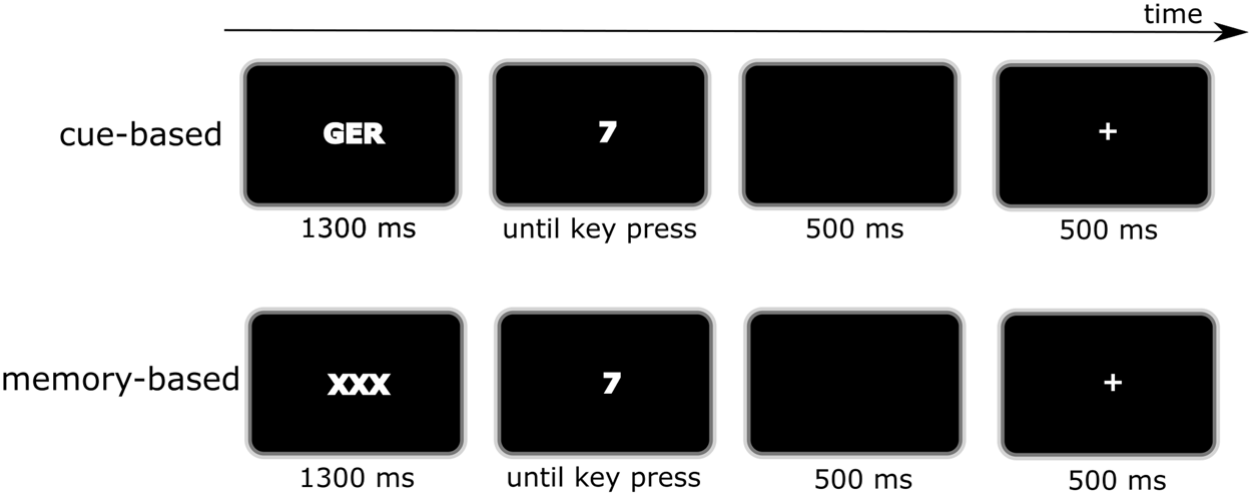
Schematic illustration of the cue-based (upper part) and the memory-based task switch paradigm (lower part). The cue-based task is initiated by one of three explicit task rules (in this example “GER” (German for “Geradzahligkeit”), asking for the parity of the upcoming digit). The cue is followed by the presentation of one of eight possible digits (“1” to “9”, without “5”). Then, a response had to be given in within 500 ms with the left or right control key of a standard keyboard. After that, a feedback (“+” or “−”) was given for 500 ms until the next trial started. In the memory-based condition, the procedure of one trial was identical, except of the presentation of the task cue, which was replaced by an uninformative cue (“XXX”). Here, a fixed trial order had to be recalled, which included all three explicit task cues (AAA, BBB, CCC).

In this study, we used a well-established task switching paradigm^24,25,34,69^. The paradigm consisted of two trial types: cue- and memory-based trials. The paradigm is illustrated in Fig. 5. The whole experiment consisted of three blocks. Every block consisted of 396 trials, which were divided into 198 cue-based trials and 198 memory-based trials. In each trial, one of eight white digits (“1”, “2”, “3”, “4”, “6”, “7”, “8”, “9”) was presented on a black background of a 20 inch CRT monitor. The digits were presented 3 mm above a white fixation cross (10 mm diameter) in two different sizes; small (7 × 10 mm) or large (12 × 18 mm). There were three task rules: According to the ‘numeric’ task rule, the requested decision was whether the digit was smaller or larger than 5. According to the ‘parity’ task rule, the requested decision was whether the digit was an odd or an even number. According to the ‘font size’ task rule, the requested decision was whether the digit was written in small or large font. A standard PC keyboard served as response device. Both the left and right control key (Ctrl buttons) were used for responses. Responses indicating “smaller than 5”, “odd number”, or “small font size” were given by pressing the left Ctrl key. The other responses, i.e. “higher than 5”, “even number”, or “large font size” were given by pressing the right Ctrl key. This is a central experimental aspect in the current study, as response-based interference processes play an important role in task switching^45^, especially when there are ‘bivalent responses’ and an overlaps in ‘response sets’. This is induced by the applied stimulus-response mapping, because the response categories “numeric”, “parity” and “font size” overlapped; i.e. left and right hand responses were required in each of these categories. The stimuli and response mappings were presented in ‘cue-based’ and ‘memory-based’ task types.

Each trial began with the presentation of the fixation cross, which was followed by one of three possible explicit task cues (1300 ms before the target) in the cue-based task, or the dummy cue in the memory-based task. The task cue was presented in white letters 3 mm below the fixation cross. The task cue “NUM” (German abbreviation for “Numerisch”, Engl. “numeric”) represented the question “Is the presented digit smaller or greater than 5?”. The task cue “GER” (German abbreviation for “Geradzahligkeit”, Engl. “parity”) represented the question “Is the presented digit odd or even?”. And the task cue “SG” (German abbreviation for “Schriftgröße”, Engl. “font-size”) indicated the question “Is the digit presented in small or large font-size?”. The three presented task cues were only presented in the cue-based task. In each trial, the cue remained visible during the following presentation of one of the eight possible digits (the target). Participants were asked to respond within a period of 2500 ms. Once the response was executed, the screen turned black for 500 ms, until a feedback stimulus was displayed for 500 ms: A plus sign indicated a correct response while a minus sign an incorrect response. The cues in the cue-based task were presented in a randomized order. Due to the randomization, participants were forced to repeat a task set (maximally four repetitions) or switch to another task set.

In the memory-based task, the cue was replaced by a dummy cue “XXX” (3 mm below the fixation cross), which was shown in every trial. The dummy cue carried no information but is important to keep the visual setup comparable for both tasks. In the memory-based task, the participants were instructed to always follow the same memorized order of task rules of 3 × “NUM”, 3 × “GER”, and 3 × “SG” i.e.{NUM, NUM, NUM, GER, GER, GER, SG, SG, SG, NUM, NUM, NUM, GER, …}). Hence, participants had to use information from working memory to recall which of the possible rules has to be applied at a given trial. This manipulation refers to Baddeley and Hitch’s^70^ working memory framework. During switching tasks, in which external cues are absent, subjects have to use internal prompts such as inner speech which help to retrieve currently relevant response sets^71,72^ while flexibly reacting on alternating visual presented targets. Thus, the need for inner articulation and flexibly responding to changing conditions are two simultaneous, but separate processes that likely interfere with each other and both demand working memory processes^73^. In case participants failed to follow this pattern of task rules for more than three consecutive trials, written rule instructions were presented on the screen. Additionally, the explicit task cues (as in the cue-based task) were presented for the next three trials to help the participant to recall the running order. This provided guidance for the participants to get back on track. Just like for the cue-based block, there was a balanced proportion of each rule (33.33%), but the frequency of switching was reduced to 33.3% due to the fixed order of task rules in the memory block. It may be argued that the cue-based and the memory based trial conditions differ in regularity because there was a sequence of trials in the memory-based block and random trial presentation in the cue-based block. Yet, there has to be a fixed trial order in the memory-based block to invoke memory processes so that participants cannot use external cues and are thus forced to use internal prompts such as inner speech to retrieve currently relevant response sets^71,72^. Further information on the task design is given in the supplemental material.

### EEG recordings and data processing

The EEG was continuously recorded from 60 Ag/AgCl electrodes (BrainAmp, Brain Products Inc.) mounted on elastic caps (EasyCap Inc.) in equidistant positions. The ground and reference electrodes were positioned at coordinates θ = 58, φ = 78 and θ = 90, φ = 90, respectively. For the reference electrode this refers to electrode position Fpz. For the ground electrode a position between AFz, AF4, Fz, and F2. No EOG electrodes were mounted because blinks and horizontal eye movement can reliably be corrected using the electrodes of the 60 channel electrode cap setup, once ICA decomposition has been performed. The sampling rate was 500 Hz. All electrode impedances were kept below 5 kΩ. The EEG data were analyzed using the BrainVision Analyzer 2 software package (Brain Products Inc.). First, a manual inspection was performed to remove all visible technical artifacts. Then, the data was down-sampled to 256 Hz and filtered using 0.5–20 Hz band-pass IIR filters (both with a slope of 48 db/oct). Additionally, a notch filter of 50 Hz was applied. After that, an independent component analysis (ICA; infomax algorithm) was applied to remove all recurring artifacts like horizontal and vertical eye movements, blinks and pulse artifacts. Horizontal and vertical eye movements can be identified by their characteristic scalp topography and shape in the ICs. All ICs showing these artifacts were discarded (mean number of discarded ICs: 4 ± 1.2).

Then, the target-locked segments were formed using all channels for repetition and switch trials, separated for the memory-based and the cues-based trials and the different blocks. Only correct trials were included in the data analysis. All segments had a length of 1500 ms starting 300 ms prior the locking time point and ended 1200 ms thereafter. Afterwards the automated artifact rejection procedure discarded segments with (i) signal amplitudes higher than 150 μV or lower than −150 μV, (ii) periods of at least 200 ms showing lower activity than 0.5 μV, and (iii) with amplitude differences between two peaks during a time interval of 100 ms that are higher than 80 μV. The number of trials (5.6 ± 3.8) discarded in the artifact rejection procedure did not differ across trial types and blocks (p > 0.4). Following that, a current source density (CSD) transformation was carried out using the spherical Laplace operator. This procedure eliminates the reference potential. The applied parameters for this operator were n = 4 splines and m = 10 Legendre polynomials (Lambda = 1 * 10^−5^). Moreover, the procedure serves as spatial filter that helps identifying electrodes that can be analyzed for different ERP components^74^. Finally, a baseline correction procedure was applied in the period between from −200 ms until 0 ms (i.e. locking point); i.e. the mean amplitude in this interval was set to zero. After these preprocessing procedures, averages were calculated for every participant and experimental condition.

For the analyses of the ERP components (P1, N1, N2 and P3), the relevant electrode sites were identified by inspecting the scalp topographies. The P1 and N1 (mean amplitudes) were quantified at electrodes P7 and P8 in the time interval from 100 to 110 ms (for the P1) and from 150 until 160 ms (for the N1). The N2 (mean amplitude) was quantified at electrode Cz using the time interval from 270 to 290 ms. The P3 (mean amplitude) was quantified at electrodes P3 and P4 using the time interval from 320 to 460 ms. Data quantification was performed at the single subject level. The choice of electrodes and time windows was statistically validated using the procedure described previously^75,76^. This procedure validated the previously selected electrode sites and time windows.

### Residue iteration decomposition (RIDE)

The temporal decomposition was performed according to a previous study employing the same experimental paradigm^24^. Procedures applied have been used before^36,43^ and accord to the RIDE manual available on http://cns.hkbu.edu.hk/RIDE.htm. Details on the mathematical procedure of RIDE can be found in Ouyang *et al*.,^36,37^. The spatial filter properties imposed by the CSD transformation, do not violate assumptions of RIDE because this temporal decomposition algorithm makes only use of latency variability irrespective of the scalp distributions of the waveforms^37^. Using RIDE, the decomposition of the ERPs in S-cluster and R-cluster is based on the stimulus onsets and response times, respectively. The C-cluster latency is derived iteratively because the C-cluster has a variable latency over single trials and is defined as being neither fully time locked to the stimulus onset nor to the reaction times. The RIDE algorithm uses a time window function to extract the waveform of each RIDE component. For the initial setting of these time windows, the time windows are chosen in a range assumed to cover the time interval within which each of the clusters are assumed to occur^37^. For the current study, an interval between 0 and 600 ms was chosen for the S-cluster, a time window from 200 to 900 ms for the C-cluster and a time window ±300 ms around the response trigger was used for the R-cluster^37^. For further details on the method see^36,77^. For peak quantification in the obtained RIDE cluster we used the same procedure as described for the peak quantification in the original ERP data (see above). The S-cluster revealed activity in the P1 and N1 ERP time windows. The S-cluster mean amplitudes were quantified in time intervals 100 to 110 ms (P1) and 150 to 160 ms (N1). The C-cluster revealed activity in the N2 and the P3 time window. For the N2 time window the mean amplitude was quantified at electrode FCz between 530 and 550 ms for the cue-based trials and between 410 and 430 ms in the memory-based trials. The mean activity in the P3 time window was quantified at electrodes PO1 and PO2 between 450 and 500 ms. For the R-cluster the mean amplitudes in the P3 time window were quantified at electrodes P4 and PO2 between 690 and 705 ms. Then, the validation method as performed for the ERPs^76^ was applied to the RIDE data, with the S-cluster, the C-cluster and the R-cluster being treated separately. This validation procedure revealed the same electrodes as identified by visual inspection.

It is important to note that the time windows used for data quantification differ between the ERP and the RIDE-decomposed data. As mentioned, the idea behind RIDE is to extract constitutend clusters of processes that are intermingled in classical ERP components. For example, for the N2 it has repetitively been shown that it is composed of the S-cluster, the R-cluster, and depending on the precise paradigm also on the C-cluster data^39^. The same is the case for the P3, where especially the R-cluster and the C-cluster have been shown to constitute processes reflected by the P3^40,43,78^. Moreover, the RIDE algorithm accounts for intra- individual variability in the data. Together, this means, that the RIDE cluster only represents a fraction of what is typically reflected in a ERP component and this fraction is moreover corrected or accounting for intra-individual variability. Therefore, the RIDE clusters cannot or are at least very unlikely to occur in the same time windows as the classical ERP components.

### Source localization analyses (sLORETA)

We used standardized low resolution brain electromagnetic tomography^79^ (sLORETA) for the source localization analysis. This analysis was based on the RIDE clusters, because only these revealed reliable effects in line with the behavioral data (refer results section). The sLORETA allorithm provides a single solution to the inverse problem^79–81^ and it has been shown that sLORETA provides reliable results without a localization bias^81^, which has also been corroborated by evidence from EEG/fMRI and neuronavigated EEG/TMS studies underlining the validity of the sources estimated using sLORETA^75,81^. For source localization, the intracerebral volume is partitioned into 6239 voxels at 5 mm spatial resolution. Then, the standardized current density at each voxel is calculated in a realistic head model^82^ based on the MNI152 template^83^. On that basis, the voxel-based sLORETA images were compared across experimental blocks (blocks 1, 2, and 3) using the sLORETA-built-in voxel-wise randomization tests with 2000 permutations, based on statistical nonparametric mapping (SnPM). Voxels with significant differences (p < 0.01, corrected for multiple comparisons) between contrasted conditions were located in the MNI-brain www.unizh.ch/keyinst/NewLORETA/sLORETA/sLORETA.htm.

### Statistical analysis

All statistical analyses were carried out using SPSS (23.0.0.3). Repeated-measures ANOVAs were carried out using the factors “repetition-switch” and “trials (cue-based vs. memory-based)”, and “blocks (1, 2, 3)”. For the neurophysiological data the factor “electrode(s)” was included as additional within-subject factor, when necessary. The Greenhouse-Geisser correction was applied to all tests and all post-hoc tests were Bonferroni-corrected.

### Data availability statement

The datasets generated during and/or analysed during the current study are available from the corresponding author on reasonable request

## Electronic supplementary material

Supplementary information
