# Supplementary material for: On the role of the prefrontal cortex in fatigue effects on cognitive flexibility - a system neurophysiological approach
Source: Sci Rep. 2018 Apr 23;8:6395. doi: 10.1038/s41598-018-24834-w (PMC5913330; doi:10.1038/s41598-018-24834-w)
Supplement: Supplementary file 1 — Supplementary information [file 41598_2018_24834_MOESM1_ESM.doc]

**Supplemental material**

**On the role of the prefrontal cortex in fatigue effects on cognitive flexibility - a system neurophysiological approach**

Vanessa A Petruo, Moritz Mückschel, Christian Beste

*Stimuli and Task*

It may be argued that for a better comparability of the cue-based and the memory-based trials, a fixed order should have also been used in the cue-based trails as well. However, this was not done for the following reasons: Cue-based and memory-based trials reflect a *within-subject* manipulation, which is known to be prone to ‘carry-over effects’. Such carry-over effects may relate to circumstances where a strategy that is used in one experimental condition (i.e., memory-based condition) can also be applied in the other condition (i.e., cue-based condition). This means that even though the cue stimulus would have been sufficient to inform switching processes in the cue-based condition, it would have been impossible to rule out (or experimentally control) whether participants had in fact only used this cue information if a fixed trial order had been used in the cue-based block. In that case, it could not be ruled out/controlled that memory-based processes are used in addition to cue-based information. Such a ‘mixture of strategies’ would very likely have altered performance and would have confounded experimental elements of this study; that is, the involvement of working memory processes. Moreover, it could not be controlled in how far a mixed strategy is used in some phases of the experiment, while this is not the case in other phases of the experiment. Furthermore, it could not be ruled out whether a mixture of strategies was subject to inter-individual variations. This would have added unexplainable variance to the data.
